# Elevated microRNA-135a is associated with pulmonary arterial hypertension in experimental mouse model

**DOI:** 10.18632/oncotarget.16011

**Published:** 2017-03-08

**Authors:** Hyun-Wook Lee, Sung-Hyun Park

**Affiliations:** ^1^ Department of Environmental Medicine, New York University School of Medicine, Tuxedo, New York, USA

**Keywords:** microRNA-135a, biomarker, pulmonary arterial hypertension, Th2 antigen, urban particulate matter

## Abstract

Multiple causes are associated with the complex mechanism of pathogenesis of pulmonary arterial hypertension (PAH), but the molecular pathway in the pathogenesis of PAH is still insufficiently understood. In this study, we investigated epigenetic changes that cause PAH induced by exposure to combined Th2 antigen (Ovalbumin, OVA) and urban particulate matter (PM) in mice. To address that, we focused on the epigenetic mechanism, linked to microRNA (miR)-135a. We found that miR-135a levels were significantly increased, and levels of bone morphogenetic protein receptor type II (BMPR2) which is the target of miR-135a, were significantly decreased in this experimental PAH mouse model. Therefore to evaluate the role of miR-135a, we injected AntagomiR-135a into this mouse model. AntagomiR-135a injected mice showed decreased right ventricular systolic pressures (RVSPs), right ventricular hypertrophy (RVH), and the percentage of severely thickened pulmonary arteries compared to control scrambled miRNA injected mice. Both mRNA and protein expression of BMPR2 were recovered in the AntagomiR-135a injected mice compared to control mice. Our study understands if miR-135a could serve as a biomarker helping to manage PAH. The blocking of miR-135a could lead to new therapeutic modalities to alleviate exacerbation of PAH caused by exposure to Th2 antigen and urban air pollution.

## INTRODUCTION

Pulmonary hypertension (PH) is a rare disease that is defined in patients based on a mean pulmonary arterial pressure of more than or equal to 25 mmHg [1, 2]. The origins of PH remain unclear and patients have a poor prognosis and decreased life expectancy [3]. The pathogenesis of PH is complex, and multiple factors can lead to different types of PH. One of those types, pulmonary arterial hypertension (PAH), which is a Group 1 PH, is caused by defects in primary arteries of the lung. PAH is characterized by severe pulmonary arterial remodeling with smooth muscle cell proliferation, which results in right ventricular hypertrophy (RVH), increased right ventricular systolic pressures (RVSP), and finally right heart failure and death [4–6].

Our group has investigated the pathogenesis of the immune response induced PAH in mice [7]. Urban particulate matter (PM) exacerbates pulmonary arterial remodeling and increased RVSPs induced by prolonged exposure to a T helper type 2 (Th2) antigen (Ovalbumin, OVA) [7]. Furthermore, we showed that interleukin-13 (IL-13) and IL-17A are critical for the development of PAH induced by Th2 antigen and PM [8]. The incidence of PH is increased in patients with autoimmune diseases [9], and in some cases these diseases are associated with increased IL-13 [10] and IL-17A [11]. We also demonstrated that neutralization of IL-13 and IL-17A prevent the development of PAH [8], however, the down-stream molecular mechanisms are unclear. In this study, to further understand PAH development, we examined the epigenetic mechanisms by which exposure to Th2 antigen and urban PM exacerbates the PAH phenotype. To evaluate a specific molecular mechanism, we focused on miR-135a.

MicroRNA (miRNA, or miR) expression is one major mechanism of epigenetic regulation. miRNAs regulate gene expression by suppressing the translation of target mRNAs, and have a role in PAH [12]. Recent reports have identified additional miRNAs that could have critical roles in cardiovascular diseases, including PH [13]. PAH, like asthma such as inflammatory disease, is a disease of the airways including airway obstruction and allergic inflammation. Allergic inflammation includes elevated total allergen-specific IgE levels and increased numbers of eosinophils and Th2 cells that secrete IL-4, IL-5, IL-6, IL-9, and IL-13 [14]. In a previous study, we screened lung tissues for miRNAs that are regulated in mice that had developed the PAH induced by exposure to Th2 antigen and PM (OVA & PM) [8]. We found that miR-135a levels were significantly increased in the lungs of OVA & PM exposed mice. Our data are further supported by other work indicating that miR-135a was increased in the lungs of antigen-exposed, sensitized mice [15]. We also showed that miR-135a levels returned to near baseline levels by neutralizing IL-13 and IL-17A [8]. Furthermore, we examined the expression of computationally identified miR-135a targets that could have a significant role for PAH. We found that bone morphogenetic protein receptor type II (BMPR2) is a target of miR-135a. In humans, mutations and the dysfunction of BMPR2 are a cause of PH [16–19]. Besides mutation of BMPR2, down-regulation of BMPR2 expression is associated with the pathology in hypoxic PH [20].

Therefore we hypothesized that up-regulation of miR-135a is a critical event for PAH induced by exposure to combined Th2 antigen and urban PM. Reducing miR-135a by AntagomiR-135a attenuated and prevented developing PAH induced by OVA & PM in this study.

## RESULTS

### miR-135a expression levels are a potent biomarker for PAH

**Figure 1:**
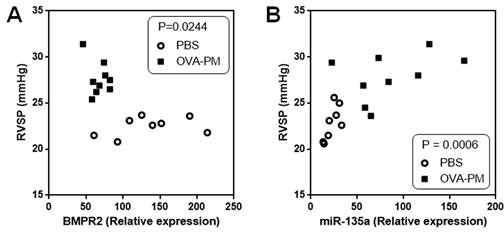
Correlation between BMPR2 expression and RVSP, and correlation between miR-135a expression and RVSP **A**. RVSP and BMPR2 expression are inversely correlated among mice exposed to PBS (*n* = 8) and mice exposed to OVA & PM (*n* = 9). **B**. RVSP and miR-135a expression are significantly correlated among mice exposed to PBS (*n* = 8) and mice exposed to OVA & PM (*n* = 9).

Because down-regulation and mutation of BMPR2 are critical in the pathology of PAH, we measured BMPR2 expression in lung of mice exposed to OVA & PM. RVSPs and BMPR2 expression are inversely correlated among mice exposed to PBS and mice exposed to OVA & PM (Figure 1A). Furthermore, because BMPR2 is a target gene of miR-135a, we measured miR-135a expression levels in lung of mice exposed to OVA & PM. RVSPs and expression of miR-135a are significantly correlated among mice exposed to PBS and mice exposed to OVA & PM (Figure 1B).

### Injection of antagomiR-135a decreases the PAH phenotype

**Figure 2:**
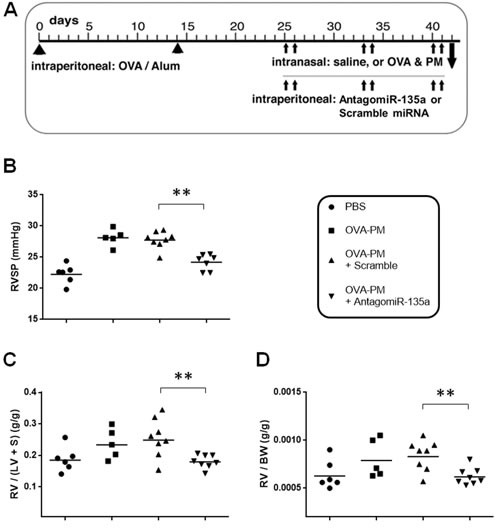
Decreased RVSP and RVH in AntagomiR-135a injected mice exposed to OVA & PM **A**. Schematic representation of the experimental schedule for sensitization and challenge by intraperitoneal injection of OVA-Alum; intranasal administration of saline (PBS) or antigen and PM (OVA & PM); and intraperitoneal injection of AntagomiR-135a or scrambled miRNA.Scatter plots show individual data points and geometric mean. RVSP is shown in **B**. The ratio of weight of right ventricle (RV) to the weight of left ventricle (LV) and septum (S) is shown in **C**. as an index of RVH. The ratio of right heart weight to body weight (BW) is also shown in **D**. Data were pooled from two independent experiments (*n* = 5 to 8 per group). Statistical analysis was performed with the unpaired, two-tailed Mann-Whitney U test. *P* value <0.01 (**) indicates significant differences between AntagomiR-135a injected mice exposed to OVA & PM and control scrambled miRNA injected mice exposed to OVA & PM.

The sensitization and challenge protocol for mice was shown in Figure 2A. We measured RVSPs using right heart catheterization [21]. Our data showed that AntagomiR-135a injection significantly decreased RVSPs in mice exposed to OVA & PM compared to control scrambled miRNA injected mice exposed to OVA & PM (Figure 2B). RVH was also significantly decreased in AntogomiR-135a injected mice compared to control scrambled miRNA injected mice (Figure 2C). The ratio of right heart weight to body weight (BW) was also significantly decreased in AntogomiR-135a injected mice compared to control scrambled miRNA injected mice (Figure 2D).

### Injection of AntagomiR-135a decreased severe pulmonary arterial thickening and remodeling

**Figure 3:**
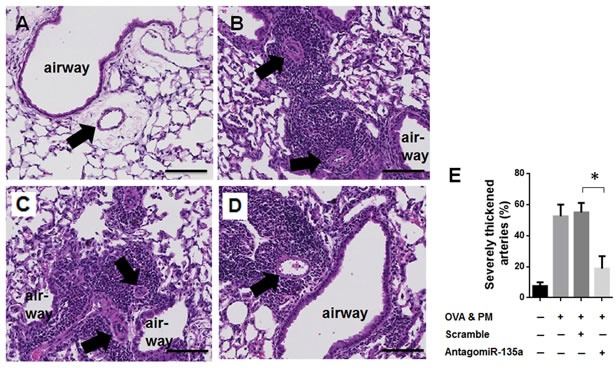
Decreased severe pulmonary arterial remodeling and thickness in AntagomiR-135a injected mice exposed to OVA & PM Photomicrographs show sections of representative of **A**. PBS exposed lungs, **B**. OVA & PM exposed lungs, **C**. control scrambled miRNA injected mice lungs exposed to OVA & PM, and **D**. AntagomiR-135a injected mice lungs exposed to OVA & PM. The sections were stained with hematoxylin & eosin (H & E). The airways are indicated and arrows point to blood vessels. Scale bars indicate 100 μm. The inflammatory infiltrates surrounding airways, blood vessels are shown in (B), (C), and (D). The bar graph **E**. shows the mean +/− SEM for the percentage of severely thickened arteries (*n* = 4 per group). Statistical analysis was performed with the unpaired, two-tailed Mann-Whitney U test. *P* value <0.05 (*) indicates significant differences between AntagomiR-135a injected mice exposed to OVA & PM and control scrambled miRNA injected mice exposed to OVA & PM.

Severe thickening and remodeling by smooth muscle cells proliferation were previously shown in mice exposed to OVA & PM [8]. Our data showed that severe pulmonary arterial thickening and remodeling were significantly increased in OVA & PM exposed mice (Figure 3B) compared to PBS exposed mice (Figure 3A). AntagomiR-135a injection significantly decreased severe pulmonary arterial thickening and remodeling in mice exposed to OVA & PM (Figure 3D) compared to control scrambled miRNA injected mice exposed to OVA & PM (Figure 3C). Severe inflammatory infiltrates surrounding airways and blood vessels are still noted in AntagomiR-135a injected mice exposed to OVA & PM (Figure 3D). However in contrast to pulmonary arterial thickening and remodeling in mice exposed to only OVA & PM (Figure 3B) or control scrambled miRNA injected mice exposed to OVA & PM (Figure 3C), Figure 3D exhibits significantly reduced arterial thickening.

### Injection of AntagomiR-135a recovered BMPR2 expression in both mRNA and protein level

**Figure 4:**
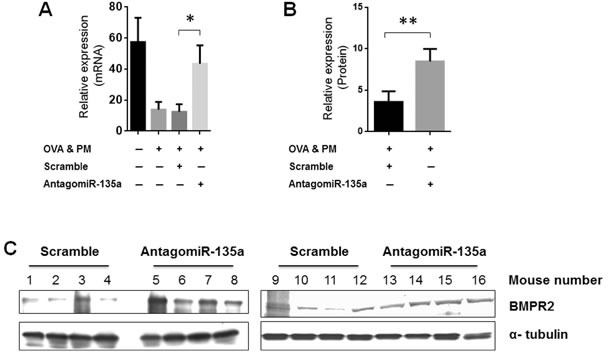
Recovered BMPR2 expression in AntagomiR-135a injected mice exposed to OVA & PM The bar graph shows the mean and standard error of the mean for BMPR2 expression in **A**. mRNA level (*n* = 4-8 per group) and **B**. protein level (*n* = 8 per group). Statistical analysis was performed with the unpaired, two-tailed Mann-Whitney U test. *P* value <0.05 (*), <0.01 (**) indicates significant differences between AntagomiR-135a injected mice exposed to OVA & PM and control scrambled miRNA injected mice exposed to OVA & PM. **C**. The mouse lung lysates were blotted with antibody to BMPR2 and α-tubulin in group of control scrambled miRNA injected mice exposed to OVA & PM and AntagomiR-135 injected mice exposed to OVA & PM. The quantitative data of protein expression was shown in (B).

BMPR2 is a target of miR-135a. We measured BMPR2 expression in AntagomiR-135a injected mice exposed to OVA & PM. Our data showed that injection of AntagomiR-135 recovered significantly BMPR2 expression in both mRNA (Figure 4A) and protein (Figure 4B and 4C) level compared to control scrambled miRNA injected mice.

### Inflammation (airway/interstitial) and the expression of cytokines in AntagomiR-135a injected mice exposed to OVA & PM

**Figure 5:**
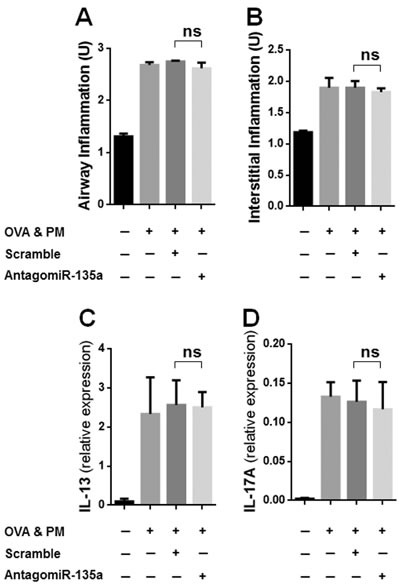
Inflammation (airway/interstitial) and the expression of cytokines in AntagomiR-135a injected mice exposed to OVA & PM The bar graph shows the mean +/− SEM for scores for airway (peribronchial/perivascular) inflammation **A**. and interstitial (alveolar) inflammation **B**., for expression of IL-13 **C**. and IL-17A **D**. in the lung (*n* = 4 per group). Statistical analysis was performed with the unpaired, two-tailed Mann-Whitney U test. ns indicates not significant between AntagomiR-135a injected mice exposed to OVA & PM and control scrambled miRNA injected mice exposed to OVA & PM.

Our data showed that airway (peribronchial/perivascular) inflammatory infiltrates (Figure 5A) and interstitial (alveolar) inflammatory infiltrates (Figure 5B) were significantly increased in OVA & PM exposed mice compared to PBS exposed mice. However AntagomiR-135a injection did not reduce inflammatory infiltrates and interstitial inflammatory infiltrates in mice exposed to OVA & PM compared to control scrambled miRNA injected mice exposed to OVA & PM. Moreover, we measured mRNA expressions of IL-13 (Figure 5C) and IL-17A (Figure 5D) in the lungs of mice. Our data showed that mRNA expressions of IL-13 and IL-17A were significantly increased in OVA & PM exposed mice compared to PBS exposed mice. However AntagomiR-135a injection did not reduce mRNA expressions of IL-13 and IL-17A in mice exposed to OVA & PM compared to control scrambled miRNA injected mice exposed to OVA & PM.

## DISCUSSION

miRNAs are short noncoding RNAs (17-23 nucleotides) that mediate post-transcriptional regulation through interaction of their target gene in the 3′ UTR region. The regulation of miRNA expression has been implicated in the pathology of PAH. Recent a few reports showed dysregulated miRNA expression in PAH and its potential new therapeutic targets for the treatments of PAH. mir-204 [22] and miR-140-5p expression [23] was down-regulated in both human PAH patient and animal model of PAH [22]. Delivery of synthetic miR-204 [22] and delivery of miR-140-5p mimic [23] to the lungs of animals with PAH significantly prevented the development of PAH, respectively.

There are several reports showing that miR-135a is involved in the pathology of lung and heart disease such as asthma or cardiac hypertrophy. The miR-135a expression was increased in allergen-challenged mouse lungs in experimental asthma model [15]. Decreased cardiac myosin binding protein C (MYBPC3) by miR-135a resulted in hypertrophy of the muscle and heart failure [24]. PAH and cancer share some common important cellular phenotypes [25, 26]. Common cellular phenotypes between pulmonary vascular remodeling observed in PAH and cancer are characterized by hyper-proliferation, resistance to apoptosis, mitochondrial dysfunction, inflammation, genomic instability, and expression of cancer biomarkers [25, 26]. Therefore miRNAs associated with cancer may be also considerable in PAH pathogenesis. Based on several recent reports, miR-135a is considered to be oncogenic. The miR-135a is up-regulated in cancer cells and tissues, and increased expression of miR-135a promotes cells proliferation [27] and cell growth [28]. Up-regulation of miR-135a was found in colorectal adenomas and carcinomas, and suppresses the expression of adenomatous polyposis coli (APC) which is a target of miR-135a [29]. Interestingly, APC was reduced in pulmonary arterial endothelial cells from PH patient lungs, so decreased APC expression is associated with the cause of PH [30]. Despite the implications of miR-135a in other diseases, the role of miR-135a is still unclear in PAH.

Our previous data showed that miR-135a expression was up-regulated in experimental PAH mice models exposed to OVA & PM [8]. BMPR2 is a target gene of miR-135a [31, 32]. Down-regulation and mutation of BMPR2 are critical in the pathology of PAH. Nonetheless, BMPR2 has not received attention as a direct therapeutic target because whether BMPR2 restoration improves PAH is still controversial. One study showed that BMPR2 restoration in rats does not improve PAH [33], whereas another study showed that delivery of BMPR2 attenuates PAH [34]. These data suggest that BMPR2 restoration alone is not sufficient to improve PAH in certain animal models with different design of BMPR2 overexpression and delivery methods or in different model systems of PH. However, our data showed that delivery of AntagomiR-135a recovered BMPR2 expression, which was down-regulated by exposed to OVA & PM. Injection of AntagomiR-135a also attenuated PAH phenotypes (RVSP, RVH, and severe pulmonary arterial remodeling) in mice exposed to OVA & PM. Our data imply that the role of AntagomiR-135a is a not only restoration of BMPR2 expression, but also has other functions to improve PAH, since miR-135a also regulates other putative target genes.

Our studies have shown that prolonged co-exposure with Th2 antigen and PM regulates microRNA expression with up-regulation of miR-135a. These changes are correlated with PAH phenotype induced by OVA & PM. Reducing miR-135a by AntagomiR-135a attenuated PAH phenotypes induced by OVA & PM. Our study is expected to advance our understanding of the molecular mechanism by which particulate urban air pollutants exacerbate immune response induced PAH. Specially, miR-135a could serve as biomarkers helping to manage PAH. Furthermore, our studies have the potential to identify new molecular targets that could be useful for therapeutic intervention in PAH and help to understand the epigenetic changes induced by Th2 antigen and air pollutants.

### Study limitations

Because of the inherent limitations of animal experimentation, our study will require follow-up work to investigate the relevance of miR-135a both in other experimental models of PAH and in human PAH. Measuring elevated level of miR-135a in blood of humans with PAH would be a powerful tool for a diagnosis. Our study has clinically translatable implications by identifying a therapeutic target miR-135a for PAH. Further translation of the findings from our study is dependent on the identification of BMPR2 down-stream genes and other target genes regulated by miR-135a. This would be a necessary step toward evaluating miR-135a as a potential therapeutic target for the clinical management of PAH. In addition, this would help to design studies to further understand how air pollution exposure could affect exacerbations of PAH.

## MATERIALS AND METHODS

### Mice

All animal experiments were performed according to guidelines outlined by the United States Department of Agriculture and the American Association of Laboratory Animal Care under the supervision and specific approval of the Institutional Animal Care and Use Committees at New York University. C57BL/6 mice were purchased from the Jackson Laboratory and maintained. We used only female mice because of that pulmonary arterial hypertension is observed more commonly in females than males [35]. The mice were housed under specific pathogen free conditions.

### Antigen sensitizing and challenge

Mice were sensitized and challenged with Th2 antigen and PM2.5 as previously published [8, 36, 37]. Briefly, mice were injected intraperitoneally with OVA (grade V; Sigma-Aldrich, 50 μg/dose) adsorbed to Alum (Imject Alum; Thermo Fisher Scientific, 2 mg/dose) to prime the Th2 immune response at two week intervals. Then the mice were intranasally challenged with OVA (100 μg/dose) and PM2.5 (25 μg/dose) two times each week, for a total of three weeks. Control mice were administered PBS intranasally. The experimental schedule is outlined in Figure 2A.

### Use of PM2.5

The dose of PM2.5 that we used was at half the concentration of the dose reported to induce significant airway inflammation using PM2.5 sampled in New York City [38], Beijing [39], and Baltimore [40]. When given without OVA, PM2.5 at the 25 μg/50 μL dose did not elicit significant airway inflammation or vascular remodeling [7]. In order to focus on the exacerbating role of PM2.5, we used a low dose, approximately half the concentration of PM2.5 that has been reported to elicit a significant inflammatory response, in OVA challenged mice [7]. Previous data clearly show that immunized mice that were challenged with PM2.5 given together with OVA had significantly exacerbated pulmonary arterial remodeling and increased RVSPs when compared with immunized mice challenged with OVA alone or PM2.5 alone [7].

### Injection of AntagomiR-135a

AntagomiR-135a (miRCURY LNA™ microRNA inhibitor for miR-135a, 5-GGAAUAAAAAGCCAUA-3) and scrambled miRNA (5-ACGUCUAUACGCCCA-3) were purchased from Exiqon. Mice were injected with 100 μg of AntagomiR-135a mixed with Lipofectamin 2000 (Life technologies) in 200 μl per intraperitoneal administration for two times each week, for a total of three weeks. For the control group, scrambled miRNA was injected at the same timing and dosage.

### Right ventricular systolic pressure (RVSP)

Right heart catheterization was performed on anaesthetized, spontaneously breathing mice via the jugular vein using established methods as previously published [21]. Mice were anaesthetized with Avertin (tribromoethanol; 250 mg/kg intraperitoneal injection). The right ventricular pressure data were analyzed using the LabChart 7 program (ADInstruments). After right heart catheterization, tissues were harvested.

### Right ventricular hypertrophy (RVH)

The right heart and left heart with septum were removed and weighed. The ratio of weight of right ventricle to the weight of left ventricle and septum was determined as an index of RVH. The ratio of right heart weight to body weight was also calculated.

### Severe pulmonary arterial thickening

Lungs were stained with hematoxylin and eosin (H&E). Severe pulmonary arterial thickening and remodeling was determined by scoring as previously described [8, 36, 37]. Stained lung sections were examined with a light microscope at 400× magnification. 20 consecutive view fields of arteries with a diameter of < 100μm were measured. The percentage of severe arterial thickening was calculated by the following formula: number of severely remodeled arteries / number of total arteries examined × 100.

### Airway (peribronchial, perivascular) and interstitial (alveolar) inflammation

Hematoxylin-eosin stained lung sections were scored for airway and interstitial inflammation as previously described [8, 37, 41]. Airway (peribronchial, perivascular) inflammation was be scored on 20 or more consecutive view fields: 1, normal with very few inflammatory cells; 2, scattered inflammatory cells up to two rings in depth; and 3, cuffs of inflammatory cells measuring three rings or more in depth. Interstitial (alveolar) inflammation was scored on 20 or more consecutive view fields: 1, normal; 2, increased numbers of cells within the alveoli; and 3, consistent increase in the numbers of cells within the alveoli, appearance of multinucleated giant cells, and thickening of the alveolar septa.

### Quantitative RT-PCR

Total RNA from lung tissue was isolated and reverse transcribed. Real time PCR was performed with 20 ng of cDNA using the Applied Biosystems 7900HT Fast Real-Time PCR system. The PCR for detecting BMPR2 was carried out with primers as follows: the forward primer of 5′AGA GGC CCA ATT CTC TGG AT3′ and the reverse primer of 5′GGA GAT GAC CCA GGT GGA C3′. The PCR for detecting β-actin was carried out with primers as follows: the forward primer of 5′GGC TGT ATT CCC CTC CAT CG3′ and the reverse primer of 5′CCA GTT GGT AAC AAT GCC ATG T3′. The following conditions were used: 95°C for 10 min, followed by 40 cycles of 95°C for 15 s and 60°C for 1 min, followed by a hold at 4°C. Raw data was then analyzed with SDS Relative Quantification Software version 2.3 (Applied Biosystems) to determine cycle threshold (Ct). Mean Ct value was determined and standardized for β-actin.

### Western blot analysis

Western immunoblotting was carried out as previously described [42]. Briefly, lung tissues were sonicated in RIPA lysis buffer with protease inhibitors. Equal amounts of protein lysates were used for SDS-PAGE analysis followed by immunoblotting. Detection methods were carried out using either Immobilon Western Chemiluminescent HRP substrate (Millipore) or WesternDot^®^ 625 Goat Anti-Mouse Western Blot Kit (Thermo Fisher Scientific).

### Statistical analysis

Statistical analysis was performed with the Prism 6 (Graphpad) software. Two group comparisons were conducted with the unpaired, two-tailed Mann-Whitney U test or the unpaired, two-tailed t-test with Welsh's correction for unequal variances. A *P* value <0.05 was considered to be significant.

## Abbreviations

BMPR2: bone morphogenetic protein receptor type II
IL: interleukin
miRs, or miRNAs: microRNAs
OVA: Ovalbumin
PM: particulate matter
PH: pulmonary hypertension
PAH: pulmonary arterial hypertension
RVH: right ventricular hypertrophy
RVSP: right ventricular systolic pressure
