# Correction: Elevated microRNA-135a is associated with pulmonary arterial hypertension in experimental mouse model

**DOI:** 10.18632/oncotarget.20578

**Published:** 2017-08-29

**Authors:** Hyun-Wook Lee, Gabriele Grunig, Sung-Hyun Park

**Affiliations:** ^1^ Department of Environmental Medicine, New York University School of Medicine, Tuxedo, New York, USA

**This article has been corrected:** Dr. Gabriele Grunig was added to the author list.

The authors sincerely apologize for this oversight.

Original article: Oncotarget. 2017; 8:35609-35618. https://doi.org/10.18632/oncotarget.16011

